# Effectiveness and Evidence Level of Dance on Functioning of Children and Adolescents with Neuromotor Impairments: A Systematic Review

**DOI:** 10.3390/ijerph20021501

**Published:** 2023-01-13

**Authors:** Elisangela F. Lima, Beatriz H. Brugnaro, Nelci Adriana C. F. Rocha, Silvia L. Pavão

**Affiliations:** 1 Department of Prevention and Rehabilitation in Physical Therapy, Federal University of Paraná, Curitiba 80060-000, Brazil; 2 Child Development Analysis Laboratory (LADI), Department of Physical Therapy, Federal University of São Carlos, São Carlos 13565-905, Brazil

**Keywords:** dance, neuromotor impairments, intervention, children, functionality

## Abstract

Objectives: The aim of this paper is to investigate the effects of dance therapy in children with neuromotor impairments (CNI), organizing the outcomes according to International Classification of Functioning Disability and Health (ICF) domains, and to investigate if there is adequate evidence of effectiveness to recommend dance as a therapy. Methods: Electronic searches were conducted in December 2021. We include studies assessing the effects of dance in CNI up to 18 years. Data extraction included studies’ populations, intervention features, and main outcomes. We classified outcomes according to the ICF framework. We used the Cochrane collaboration’s tool, modified by effective practice and organization of care (EPOC), to assess the methodological quality. The GRADE synthesized the body of evidence. Results: Twelve studies were included, with most of them addressing the body structure and function and activity components of ICF. Only three studies addressed components of participation, and four of personal factors. All these studies reported the positive effects of dance. Nevertheless, all of them presented high risk of bias. We found very low evidence level for improvement of body structure and function and activity components. Conclusion: Dance therapy presents low evidence level for improvements of body structure and function and activity in CNI. Further studies with low risk of bias and larger samples are needed.

## 1. Introduction

Health conditions with structural damages to the central nervous system may result in multiple neuromotor impairments, which differ in their severity, according to the degree and location of the lesion [1,2]. Children with neuromotor impairments present deficits in posture and movement, which may impact their activity and participation level, constraining their social function, engagement in tasks, and performance of physical activities [3,4,5].

According to Field and Roxborough [1,2] conditions such as, cerebral palsy, developmental coordination disorders, myelomeningocele, chronic syndromes with motor impairments, and neurodegenerative diseases could be considered neuromotor impairments. Children with neuromotor impairments often present neuromuscular alterations, deficits in postural control, and inability to perform simultaneous tasks [6,7]. Therefore, due to their health condition, children with neuromotor impairments commonly need therapeutic interventions to deal with their dysfunctions and maximize their function [8,9,10].

The aim of different therapies is promoting coordination, improvements in posture, muscle strength, motor learning, and executive functioning, in order to improve participation [11,12] and functionality [5,6,7,8]. Therefore, the adopted therapies should preferably be based on a biopsychosocial model of health, in order to promote functionality in multiple health domains [3,13,14].

In the context of the biopsychosocial health model, the International Classification of Functioning, Disability, and Health (ICF), published by World Health Organization, considers functioning as the result of multidirectional relationship between positive aspects of body structures and functions, levels of activity, and participation, and it may be influenced by contextual factors, such as personal and environmental issues [3,14,15,16].

Based on the ICF framework, rehabilitation programs directed to children and adolescents with neuromotor impairments should not only focus on the components of body structure and function, such as increases in muscle strength, range of movement, and postural stability, but also be directed to improve performance in activities of daily living and engagement in activities in home, school, and community environments [7,9].

Among current available therapies presenting potential to act in all these health domains, we find in dance a therapeutic resource with great potential for these multiple biopsychosocial components, which would be awakened by the simple act of dancing [9]. As a therapeutic resource, dance intervention is the training of a neuromotor activity based on repetition and sequences of ordinary and dynamic movement memorization to music [5,17]. According to Lakes et al. [5] and Withers et al. [9], dance’s therapeutic benefits go beyond conventional physical therapy. They report that dance may act on self-confidence, allowing us to use the body as an instrument of creative expression, beyond exposing it to music and rhythm to guide movement. Dance also acts as an alternative structure for physical rehabilitation interventions, potentially improving not only physical and cognitive functions, but also sociocultural interaction and emotional well-being [5]. Therefore, dance might be a resource in the search for consistent alternatives to increase the quality of life and social integration of young people and children with neuromotor impairments [18,19,20].

In a recent systematic review investigating the use of dance and movement with music (rhythmic auditory stimulation (RAS)) in the neurorehabilitation of children and adults with cerebral palsy, López-Ortiz et al. [21] reported preliminary evidence of the benefits of dance and RAS on body functions, particularly balance, gait, walking, and cardiorespiratory fitness, for this population. The authors pointed out the absence of studies addressing the effects of dance in the activity and contextual domains of ICF. Nevertheless, the authors’ investigation was limited to cerebral palsy, without search for dance effects on other health conditions that might constrain neuromotor functions [1].

Considering the multiple physical, psychological, and social aspects addressed by dance, as well as the playful and positive involvement aspects aroused by the act of dancing [5,9], we consider that the rhythmic movements performed to music, which characterize dance, would be a therapeutic resource to be used in rehabilitation programs possibly presenting positive effects in multiple health domains of children and adolescents with neuromotor impairments. Nevertheless, we currently do not know: (1) what are the effects of dance on the different health domain of these individuals; (2) if dance would be adopted as a therapeutic intervention for this population with enough level of evidence.

Therefore, we systematically reviewed literature of clinical trials addressing the effects of dance as the main rehabilitation resource in children with neuromotor impairments, organizing the used outcome measures according to the ICF domains, aiming to report dance effects on each of these addressed domains and investigate if there is adequate evidence of effectiveness to recommend dance as a therapy for these children.

This investigation allows not only to report the level of evidence of dance as a therapeutic tool in neuromotor disabilities, but also to outline its impact on the functionality of children addressing different health domains, thus adopting a biopsychosocial model of health.

## 2. Materials and Methods

### 2.1. Search Strategy

The adopted protocol for this review was based on PRISMA statement checklist (PRISMA) [22]. It was registered on the PROSPERO website (https://www.crd.york.ac.uk/prospero/—CRD42020212876, accessed on 4 March 2022). The protocol included the effects of rehabilitation based on dance therapy in children with neuromotor impairments. In this review, we only used data related to therapies involving dance as main therapeutic resource.

### 2.2. Identification and Selection of Studies

Two independent reviewers (E.F.L. e S.L.P.) performed a search on the following databases: PubMed, Web of Science, Embase e Scopus, from the oldest search date of each database until 22 December 2021. We used the following string: (child[Title/Abstract] OR children[Title/Abstract] OR adolescent[Title/Abstract]) AND (dance[Title/Abstract] OR “dance therapy”[Title/Abstract] OR “dance rehabilitation”[Title/Abstract]) Filters: Full text ((“child”[Title/Abstract] OR “children”[Title/Abstract] OR “adolescent”[Title/Abstract]) AND (“dance”[Title/Abstract] OR “dance therapy”[Title/Abstract] OR “dance rehabilitation”[Title/Abstract])) AND (fft[Filter]). Language restrictions were applied to include only articles in English.

The reviewers selected the studies for this systematic review according to inclusion criteria described below. In the case of disagreements, a third reviewer would resolve discrepancies. We adopted the following steps: first, we excluded duplicate studies; second, we selected studies based on titles and abstracts according to the inclusion criteria; then, we selected these previous articles after reading the full text. Moreover, we conducted a manual search in the reference lists of the included studies to confirm the existence of other studies that were not initially tracked. We used the State of the Art through Systematic Review (START), version 3.0.3 BETA, software for study selection [23].

To define the inclusion criteria, the participants, interventions, comparisons, outcome, and study (PICOS) design model was applied.

### 2.3. Population

Selected studies should include children with neuromotor impairments, from both sexes, aged 0 to 18 years. According to Field and Roxborough [1] and Pena et al. [6], we considered children with neuromotor impairments the ones with medical conditions such as cerebral palsy, developmental coordination disorders, myelomeningocele, chromosomic syndromes with neuromotor impairments, and neurodegenerative diseases.

### 2.4. Intervention

The intervention should include protocols involving dance as main therapeutic resource for these children’s rehabilitation. Dance therapy was considered the systematic training of a neuromotor activity based on repetition and sequences of ordinary and dynamic movement memorization to music [5,17]. Incorporating dance as an art form into rehabilitation has capacity to transcend traditional barriers in therapy that differentially focus on impairments and limitations [9].

### 2.5. Comparators

The comparators were traditional therapy, or the subjects themselves, since we also included before–after studies without an external control group.

### 2.6. Outcome Measures

We included all types of outcome measures addressed by the studies, since one of the aims of our review was to identify the measured outcomes used in the studies and classify them according to ICF domains.

### 2.7. Study Type

Eligible study designs included randomized, quasi-randomised controlled clinical trials, controlled before–after studies, and before–after studies without an external control group. The inclusion of these multiple study designs was an attempt to provide a broader overview of this therapeutic resource (dance), considering its immense biopsychosocial potential [24].

### 2.8. Data Extraction

After selection, the following data were extracted from the included studies: (1) study design; (2) participants characteristics (sample size, age range, neuromotor impaired studied group, adopted classifications), according to medical condition; (3) characteristics of intervention (total minutes of dance intervention’s session, frequency of intervention, total duration of total intervention, adopted intervention for control group, and follow up); (4) classification of outcome measures according to functionality components present in ICF framework; (5) effectiveness of dance intervention in each of the addressed ICF domains; (6) methodological quality assessment of the studies; (7) evidence synthesis of dance as a therapeutic resource for children with neuromotor impairments.

### 2.9. Methodological Quality Assessment

We assessed the methodological quality of the studies using the Cochrane Collaboration’s tool, modified by EPOC24-28, for assessing risk of bias, which evaluates: (1) random sequence generation, (2) allocation concealment, (3) baseline characteristics similar, (4) baseline outcomes similar, (5) confounding unlikely, (6) appropriate analyses (e.g., adjusted or time trend analyses if required), (7) sample representative of source population, (8) intervention independent of other changes, (9) intervention integrity (e.g., intervention delivered as intended and consistently, adequate protection against contamination), (10) blinding of outcome assessment, (11) incomplete outcome data addressed, and (12) free of other bias. The studies were evaluated independently by two reviewers (E.F.L. and S.L.P.). We used the kappa index to measure agreement between the researchers (inter-rater reliability). The kappa index was 0.98 (*p* < 0.001). In case of disagreement, a third reviewer would be consulted to reach a final decision.

### 2.10. Evidence Synthesis

Aiming to perform an evidence synthesis, we grouped the studies according to the outcome measures. Beyond the reporting of evidence synthesis for measured outcomes, we presented these results according to ICF domains in which they were previously classified, and then we should also express existent evidence level according to ICF domains.

For this review, we considered a minimum of 2 studies per measured outcome. Studies that could not be grouped were excluded for the evidence synthesis. The evidence synthesis was performed using the grading of recommendations assessment, development and evaluation (GRADE) approach, considering the following domains for analysis: risk of bias, inconsistency of results, indirectness, imprecision, and publication bias. The level of evidence was classified as: high (enough evidence in the estimate of the effect), moderate (the true effect is close to the estimate of the effect), low (the confidence of the effect is limited), and very low (little confidence of the effect estimate), according to GRADE system [25]. A strong or moderate level of evidence represented a strong recommendation for use of the intervention.

## 3. Results

### 3.1. Search Strategy

**Figure 1:**
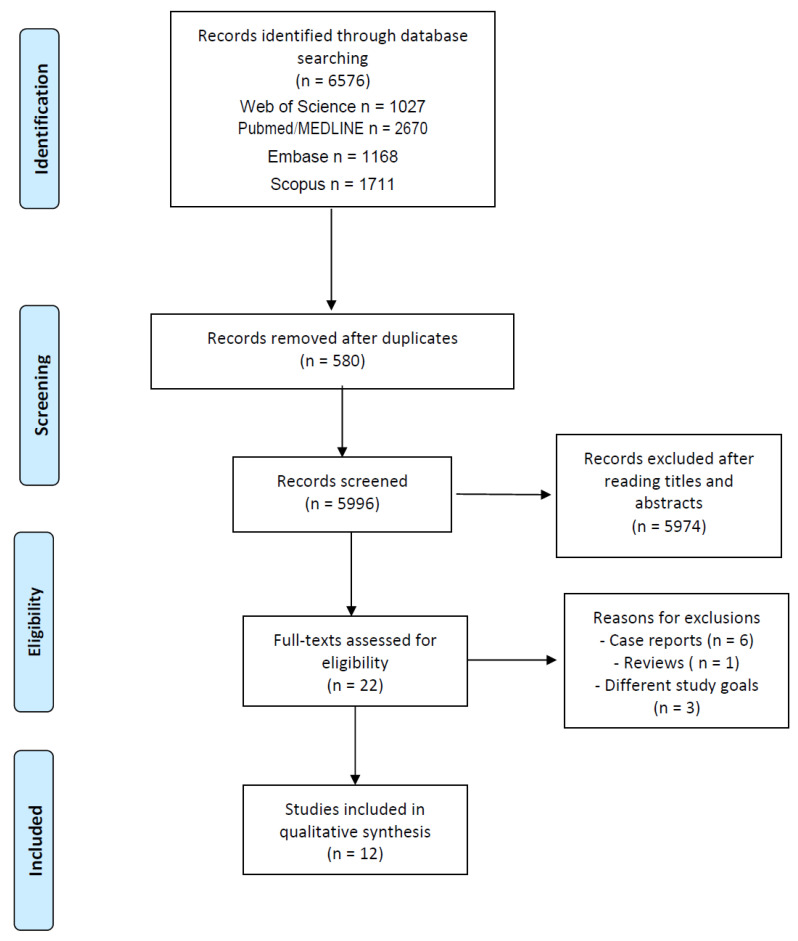
Information flow diagram of the phases of the systematic review, based on PRISMA protocol (n = number of studies).

The search strategy yielded 5996 articles. Twelve articles met the full inclusion criteria. The excluded studies and reasons for exclusions are presented in Figure 1.

### 3.2. Participants and Intervention Characteristics

We included studies which aimed to investigate the effects of dance as a therapeutic resource in children with neuromotor impairments up to 18 years. Seventy percent of the studies evaluated dance effects on children with cerebral palsy [5,9,17,26,27,28,29]. The remaining 30% evaluated children with Charcot Marie Tooth [30], Willians syndrome [31], Down syndrome [32], and extremely preterm children with motor impairment [33].

Cerebral palsy was the most studied health condition (n = 8), with included children in all gross motor functions levels, according to the gross motor function classification system (GMFCS) [5,9,17,26,27,28,29,33]. 

Dance was the only evaluated therapeutic resource for intervention groups in all included articles. The frequency of intervention varied 1–3 times/week, with therapeutic sessions from 30–120 min. Protocol duration varied between 6 and 12 weeks. Only Withers et al. [9] adopted a 20-month protocol. Follow up was adopted by only two studies: Lakes et al. [5], five weeks, and López-Ortiz et al. [28], one month. Table 1 shows the description of the evaluated groups and characterizes the adopted intervention.

### 3.3. Outcome Measures According to ICF Domains

Classifying outcome measures according to ICF domains, from the 12 included studies in this review, 7 studies adopted outcomes addressing body structures and function ICF components [5,9,17,26,27,30,31,33], 11 addressed activity component [5,9,26,27,28,30,31,32,33,34], 3 addressed participation components [29,32,33], and 4 contextual components, with all of them addressing personal factors [9,27,31,33].

Only four studies used outcome measures encompassing dance effects on functioning of children with neuromotor impairments in a broad way, using additional outcomes to those of the structure, function, and activity domains, such as participation [33] and personal factor components [9,27,31,33]. The classification of the measured outcomes on the different ICF domains is show in Table 2.

#### Effectiveness of Dance Intervention

Dance was the only therapeutic resource for the intervention groups in all included articles. Table 3 shows the main results of these studies. The results are grouped in the table according to the ICF domain in which the outcomes were previously classified.

All included articles pointed out dance as an effective therapeutic resource to improve the components of body structure and function in children with neuromotor impairments [9,26,27,31,32]. Significant results were reported for strength improvements of the hip, knee, and ankle muscles, increases in the range of movement of the hips and ankles, pain reduction during physical activity, and decreasing in standing postural sway.

Still, all the studies addressing activity components reported significant gains in this domain [5,9,17,26,27,28,30,31,32,33,34], with improvement in functional balance, cognition (performance of rhythmic and attention tasks, increasing in gait velocity and cadence, step length, postural stability, postural transfers, functional independency, locomotion, basic mobility, self-care, psychosocial adjustments, communication, coordination, agility, and global, sports and physical functions).

Participation gains were observed, addressing parents and therapists’ perceptions of greater engagement of the children on social, physical, and therapeutic activities [29,32,33].

Positive results were also found in personal factors. The authors reported improvement in body image acceptance, increased level of happiness, sociability, reduction of emotional problems, increased self-confidence, and satisfaction for carrying out the therapies [9,26,31,33].

### 3.4. Methodological Quality Assessment

Considering the risk of bias of individual studies, all of them were classified as high risk. Among the identified reasons for this classification, we may cite: lack of randomization, concealed allocation, differences in baseline measures, no correction for confounding, and no application of intention to treat analysis. In fact, only two studies used a randomized controlled design [17,34]. The main methods of limitations across studies were that 90% did not blind the therapists, 50% did not blind the patients, 7% did not use concealed allocation, and 3% did not perform intention to treat analysis.

The detailed methodological assessment of the studies is presented in Table 4.

### 3.5. Evidence Synthesis

Using the GRADE ‘summary of findings’ table, we found that the quality of evidence for the use of dance as a therapeutic resource for body structure and function and activity level of children with neuromotor impairments was very low. We grouped the studies for evidence synthesis based on the outcome similarities, which are shown on Table 5. The evidence synthesis is organized on the table according to the ICF domain in which they were classified. The found “very low level of evidence” was related with risk of bias, inconsistency, indirectness imprecision, and publication bias.

## 4. Discussion

The present study aimed to systematically review articles which used dance as a therapeutic resource for the rehabilitation of children with neuromotor impairments, thus investigating: the measured outcomes used by the studies, classifying these outcomes according to ICF domains; the reported effects of dance on each of the addressed ICF domains; if there is adequate evidence of effectiveness to recommend dance as a therapy for these children.

Our study design allowed us to comprehensively gather, describe, categorize, and synthesize the research literature on dance as a therapeutic resource published in English from 2012 to 2021. These data show us that, apparently, the interest in dance as a rehabilitation tool in children with neuromotor impairments is recent. This interest appears to have grown recently, as suggested by the finding that 10 from the 12 included studies were published in the last four years. Moreover, we could observe that the quality of the studies have improved recently.

Dance as a therapeutic tool, by its very nature, has the potential to impact multiple subsystems [9]. In fact, outcome measures of the included studies assessed components of ICF in the majority of health domains, as shown in Table 2. Nevertheless, none of the included studies have adopted an entire biopsychosocial approach assessing outcomes in each of the ICF components, thus comprehensively determining the effects of dance on the functionality of children with neuromotor dysfunction, according to the ICF framework.

Most of the tested outcomes are classified on the body structure, function, and activity domains. These results show us that the great researchers’ concerns seem to still be focused on interventions that modify the biological and structural aspects of these children and improve specific activities. We also noted a lesser concern to assess the effects of interventions on the participants’ level of participation and engagement in different activities and environments, as well as on personal factors, such as satisfaction and happiness. Still, there were a lack of studies addressing how dance, as a therapeutic resource, can modify the relationship of the participants with the environment around them.

Health conditions impairing neuromotor system may promote structural changes on subjects [1] which may not only be modified by physical therapy. Moreover, beyond these physical changes, subjects with neuromotor impairment might suffer from personal, environmental, and social changes, which would constraint their activity and participation, impacting their relationship with community and recreational practices [5,35,36,37], as well as their quality of life and emotional wellbeing. Therefore, aiming to improve functioning in this population, it is crucial that further randomized clinical trials include outcome measures addressing multiple health domains of the subjects.

Beneficial effects of dance as a therapeutic resource for children with neuromotor impairments were reported by most of the authors whose studies were discussed in our review. Most publications addressed children with cerebral palsy, the most common developmental disorder in childhood [38]. In comparison, the number of studies on other health conditions affecting movement, such as Down syndrome, dystrophies, and developmental coordination disorders, was relatively small. Dance shows potential benefits for these children, but there are still few studies addressing this issue.

We found positive effects in all assessed health domains, including improvement in body structure and function, activity, participation, and personal factors, which are described in Table 3. Nevertheless, all the studies were classified with high risk of bias, which natively impacted their methodological quality. The main methodological biases found were the small sample size in the studies, absence of blinding of assessors, absence of well-matched control groups, random assignment of subjects, and of intention-to-treat analysis.

Even though children with cerebral palsy were the most studied population in the articles included in our review, it was not possible to certify the evidence of the effect of dance in this specific population or for the population of children and adolescents with neuromotor disabilities in general. This impossibility is especially due to the large number of methodological biases found in the studies. In fact, we noted that only two of the included clinical trials adopted a randomized controlled design [17,34], and just another three had control groups [9,28,30].

The adopted outcome measures for each of the ICF domains were also quite variable. The heterogeneity in the design of interventions, such as session duration, frequency of sessions, and duration of the protocol, were factors that also challenged this review [39]. The prevalence of sparse descriptions of the components of interventions also made it challenging to characterize interventions precisely and thoroughly, and to compare the key components of different interventions. We are aware that the variation in dance interventions may mean that the evidence gathered is not, in fact, able to provide any consistent recommendations, as they are comparing vastly different interventions, i.e., ballet has a very different content to hip hop, and classes run by a dance teacher will be significantly different to those run by a physiotherapist. Nevertheless, regardless of the adopted dance stile in the interventions, we consider dance therapy as the systematic training of a neuromotor activity based on repetition and sequences of ordinary and dynamic movement memorization to music [5,17]. Moreover, only in the studies run by Cameron et al. [33], Lopez-Ortiz [29], and Joung et al. [27], dance sessions were not conducted by physical therapists.

These challenges notwithstanding, this review found that dance interventions for children and adolescents with neuromotor impairments are quite diverse. These multiple features in intervention designs have several implications for advancing the practice and science of dance as a therapeutic tool [40].

We found a very low level of evidence for dance as a therapeutic resource in all tested components of activity and body structure and function, as is shown in Table 5. This lower level of evidence is probably due to the few randomized controlled trials we found; moreover, most studies included small samples, varying the types of exercises and intensity in both groups.

In a recent published review addressing the use of dance and movement with music (rhythmic auditory stimulation (RAS)) in the neurorehabilitation of children and adults with cerebral palsy, Lopez-Ortiz et al. [21] found results quite different from ours. The authors reported the existence of preliminary evidence of the benefits of dance and RAS for individuals with CP. Nevertheless, we highlight some important differences between these reviews, related to the inclusion criteria stablished by PICOS design model. Complying with PICOS’ criteria, from the 11 and 12 included articles in Lopez-Ortiz et al.’s and ours review, respectively, only three studies [17,28,29] were commonly inserted in both reviews, which may explain the different conclusions found.

According to our results, evidence does support the indication of dance as a therapeutic resource to improve body structure and function, as well as activity, in children with neuromotor impairments. Accordingly, dance therapy could even be recommended with caution, if appropriate to a child’s goal with careful assessment, just as the orange light studies were recommended in that way in the reviews of interventions for children with cerebral palsy by Novak et al. [8].

The current rehabilitation framework for children with neuromotor disabilities is no longer based on the improvement of anatomic and physiological deficits [41]. The primary goals are to promote function, empower families, improve fitness, promote participation in fun activities, and enhance opportunities to develop meaningful peer connections, which has been considered an intervention that could enhance participation, while providing valuable physical exercise [41]. These findings could indicate that the effects of dance might go beyond the constructs of activity and body structure and function. Nevertheless, these effects should be further studied.

Based on our results, we recommend that further clinical trials assess the effects of dance as a therapeutic resource using outcome measures in all components of ICF. Therefore, randomized controlled studies with higher methodological quality, and analyzing a larger number of variables, are needed to draw conclusions about the effectiveness of dance as a therapeutic tool. The main results of our study are described in a illustrated infographic available in Supplementary Materials.

The main limitations of our systematic review were: (1) heterogeneity regarding the design of interventions; (2) the small sample sizes in the studies; (3) inclusion of clinical trials with non-controlled and non-randomized designs. Moreover, it would be better to not limit our search by language; nevertheless, we opted to limit to studies published in English, since it is global language. These limitations compromised the quality of the systematic review and the drawing of convincing conclusions. Some strengths of this review included the prospective registration and the duplicate data processes.

## 5. Conclusions

Besides the found positive effects of dance as a therapeutic tool, there is still a very low level of evidence for its use as an intervention to improve body structure and function and activity level in children with neuromotor dysfunction. Further randomized controlled trials with a higher methodological quality and larger outcome measures are needed to draw conclusions about the effectiveness of dance as a therapeutic tool. Further studies should assess the effects of dance in this population using outcome measures in all components of the ICF, especially on participation and personal factors.

## Figures and Tables

**Table 1:** Participants and intervention characteristics.

Study	Study Design	Participants				Intervention	Duration				
		N (IG+CG)	Age Range	Health Condition	Classification Health Condtion		Session	Frequency (Days/Week)	Protocol	Follow Up	Comparators
[33]	Case series design	N = 10 IG = 10 CG = 0	0–3 years	extremely preterm and extremely low birth weight expressing motor impairments at 3 years’ corrected age	MABC-2 scored ≤16th	Dance PREEMIE	30–60 min	1×/week	8 weeks	-	-
[34]	Randomised controlled, double-arm trial	N = 36 IG = 18 CG = 18	6–10 years	Down syndrome	<5 Beighton’s hypermobility test	Indian classical dance	60 min	3×/week	6 weeks	-	Conventional therapy
[26]	Non-randomized controlled clinical trial	N = 9 IG = 5 CG = 4	7–12 years	CMT	-	Adapted dance program	60 min	2×/week	10 weeks	-	Regular care
[30]	Case series design	N = 10 IG = 10 CG = 0	10–17 years	CP	GMFCS I a III Spastic N = 6 Ataxic N = 1 Dyscinetic N = 1 Spastic dyscinect N = 1	Dance intervention.	60 a 90 min	2×/week	10 weeks	-	-
[27]	Single-group cohort study	N = 13 IG = 13 CG = 0	13–20 years	CP	GMFCS I N = 3 GMFCS II N = 7	Let’s Be Creative Dance Exercise program	120 min	2×/week	12 weeks	-	-
[9]	Non-randomized controlled clinical trial	N = 18 IG = 9 CG = 9	10–13 years	CP	GMFCS I GMFCS II	Adapted hip-hop dancing	60 min	1×/week	20 months	-	Conventional therapy
[31]	Case series design	N = 4 IG = 4 CG = 0	5–10 years	Willians syndrome	-	Dance/ movement therapy (DMT)	60 min	1×/week	10 sessions	-	-
[28]	Non-randomized controlled clinical trial	N = 12 IG = 6 CG = 6	7–15 years	CP	GMFCS II N = 6 GMFCS III N = 3 GMFCS IV N = 2	Targeted dance class, utilizing classical ballet principles for rehabilitation	60 min	3×/week	4 weeks	After 1 month	Conventional therapy
[29]	Single-group cohort study	N = 16 IG = 16 CG = 0	-	CP	GMFCS I e II GMFCS III e IV	Classical ballet program	-	1×/week	5 a 8 weeks	-	-
[32]	Single-group cohort study	N = 14 IG = 14 CG = 0	4–13 years	Down syndrome	-	Adapted dance program	60 min	1×/week	20 weeks	-	-
[5]	Single-group cohort study	N = 8 IG = 8 CG = 0	9–14 years	CP	-	Therapeutic Ballet	60 min	3×/week	6 weeks	After 4–5 weeks.	-
[17]	Randomized controlled clinical trial	N = 26 IG= 13 CG= 13	15–29 years	CP	GMFCS II N= 9 GMFCS III N= 8 GMFCS IV N= 7 GMFCS V N= 2	Dance class	60 min	2×/week	12 weeks	-	Conventional therapy

N (sample size), IG (Interventions Group), CG (Control Group), CP (Cerebral Palsy), CMT (Charcot Marie Tooth) e GMFCS (Gross Motor Function Classification System); MBAC-2 (Movement Assessment Battery for Children-2nd edition).

**Table 2:** Classification of Outcome Measures according to ICF domains.

Study	Body Structure and Function		Activity			Participation		Contextual Factors		
	Variable	Evaluation Tool	Evaluated Task	Task Manipulation	Instruments	Evaluated Components	Instruments	Personal	Environmental	Instruments
[33]	- Motor impairment	- MABC-2	- Parents set goals for the children with a focus on motor and cognitive activity components		- COPM	- Parents set goals for children with a focus on participation	- COPM	- Enjoyment of dance classes	-	- Smiley face scale
						- Sedentay behavior and physical activity time	- Pre-PAQ			
[34]	-	-	- Agility and coordination on gait, control of object manipulation, grasping, visual-motor integration skills - dynamic balance and motor planning - functional balance		- TGMD-2 - FSST - PBS	-	-	-	-	-
[26]	- Pain	- EVA	- Stay in orthostatism	- Eyes opened, eyes closed, and dual-task condition	- Subtest of Mira Stamback - Score! and Score DT - (Tea-Ch)	-	-	-	-	-
	- Level of incapacity	- CMTPedS								
	- ROM (knee extensors; ankle dorsiflexors)	- Goniomery	- Cognitive task and rhythm							
	- Strength measures hip, knee - And ankle muscles - Muscle Power - AP and ML amplitude, velocity of CoP sway	- Lafayette Force platform		- Simple and divided sustained						
	- Memory short term and working memory - Task memory	- WISC-IV	- Task attention							
[30]	- AP and ML amplitude and velocity of CoP sway	- Force platform	- Functional balance - Manual reaching - Gait - Rithimic tasks - Attention	- Simple, sustained, divided sustained	- PBS - PRT - 10 min walking test - Subtest of Mira Stamback - TEA-Ch (Score! and Score DT (Tea-Ch)	-	-	-	-	-
[27]	- Hip, knee, and ankle ROM in sagital plane	- Vicon Motion System	- Gait - Gross motor function	-	- Velocity; cadence; step length; support time uni/bipodal - GMFM-88	-	-	- Body image	-	- BCS
[9]	- Pain and comfort level	- PODCI	- Upper extremity and physical function - Transfer and basic mobility—Sporting and physical function - Global function	-	- PODCI	-	-	- Happiness level - Sociability and emotional and behavior problems - Concentration, self-confidence, and aptitude	-	- Parents self-applicable questionnaire - PODCI - CBCL - Instructor’s perspective
[31]	- AP/ML amplitude and of sway of CoP - Strength of knee extens/flexors, ankle dorsiflexors/plantar flexors	- Force platform - Handheld dynamometers	- Static stance maintenance - Functional mobility	- Eyes open/eyes closed	- TUG	-	-	- Sociability and emotional and behavior problems	-	- CBCL
[28]	-	-	- Functional balance. - Quality of movement upper extremities		- PBS - QUEST	-	-	-	-	-
[29]	-	-	-	-	-	- Parental participation and perception of dance therapeutic benefits	- Specific questionnaires for parents, children, and therapists, based on the LIFE-H	-	-	
[32]	-	-	- Gross motor function - Motor performance	-	- GMFM-88 - COPM	- Performance, satisfaction with individualized participation goals	- COPM	-	-	-
[5]	- BMI - Percent body fat and bone density - Hand Grip Strength	- Stadiometer - Densitometer - Handgrip dynamometer	- Selective voluntary motor control - Gait - Executive function - Habitual activity level	-	- ESCALE - GAITRite - Hearts and Flowers EF tasks - Activity monitors	-	-	-	-	-
[17]	-	-	- Funtional activity - Functional independence	-	- WHODAS - FIM	-	-	-	-	-

Legend: Visual Analog Scale (VAS), Range of Movement (ROM), Anteroposterior(AP), Mediolateral (ML), Center of Pressure (CoP), Test of Everyday Attention for Children (TEA-Ch), Wechsler Intelligence Scale for Children (WISC-IV), Charcot-Marie-Tooth disease Pediatric Scale (CMTPeds), Selective Control Assessment of the Lower Extremity (ESCALA), Gross Motor Function Measure (GMFM), Timed up and Go (TUG), Canadian Occupational Performance Measure (COPM), Pediatric Balance Scale (PBS), Pediatric Reach Test (PRT), Body Carhexis Scale (BCS), Questionnaire Child Behavior Checklist (CBCL), Quality of Upper Extremity Skills Test(QUEST), Pediatric Balance Scale (PBS), Dual X-ray Absorptionmetry (DXA), Software Centers for Disease Control (CDC), Gold standard system in gait analysis (GAITRite), Hearts and Flowers EF tasks (Tasks that measure Executive Functions), Activity monitors (Actigraph GT3X), Functional Independence Measure (FIM), World Health Organization Disability Assessment Schedule (WHODAS) e International Classification of Functioning, Disability and Health (CIF), Pediatric Outcomes Data Collection Instrument, (PODCI), Assessment of life habits (LIFE-H), Child Behavior Checklist (CBCL); Test of Gross Motor Development-2 (TGMD-2); Four Square Step Test (FSST); Preschool Physical Activity Questionnaire (Pre-PAQ); Movement Assessment Battery for Children-2nd edition (MABC-2); Root Mean Square (RMS).

**Table 3:** Reported outcomes on intervention group, according to the ICF.

Study	Main Results				
	Body Structure and Functions	Activity	Participation	Contextual Factors (Personal)	Contextual Factors (Environmental)
[33]	Pre- and post-intervention using the MABC-2 were not compared.	In the COPM performance score, score, each child had at least one goal with clinically significant improvement (median of 2 goals achieved, range 1–4). Four children achieved both participation and activity goals, three children achieved goals classified as activity, while one child achieved a participation goal.	In the COPM performance score, one child achieved the goals classified as participation. Not able to compare pre- and post-intervention using the Pré-PAQ.	In the COPM performance score, all children achieved the goals classified as satisfaction.	
[34]	-	The traditional Indian dance improved the locomotor skills, objective control, dynamic balance, and motor planning of children with Down syndrome more than that of neuromuscular exercises. Both the dance and neuromuscular training equally impacted the balance capacity.	-	-	-
[26]	Improvements on hip extensors, knee flexors and ankle dorsiflexor muscle strenght. Pain levels during physucal activity decreased following the protocol.	Improvements in cognitive features (attention and rithmic tasks).			
[30]	No significant change was observed following protocol on the center of force amplitudes and speeds in the AP and ML axis for the static balance test.	PBS and PRT scores increased following dance protocol. PRT increased during first month of intervention, then remaining constant. There were increases in rhythm production.			
[27]	Hip and ankle range of movement during walking increased in sagittal plane.	Significative improvements in GMFM scores dimensions D and E, walking velocity and cadence, step and stride length. The time of opposite foot off and first double-limb support decreased, whereas percentage of single-limb support time increased following protocol.		Scores of body cathexis scale increased.	
[9]	Reduction of somatic complaints following dance intervention.	Improvements in transfer and basic mobility domains from pediatric outcomes data collection instrument, as well increase in sporting and physical function and global function following dance intervention.		Reduction in emotional and behavioral problems and an increase in social competence in the biopsychosocial profile.	
[31]	Participants showed decreased postural sway in static stance with eyes open and with eyes closed following intervention. There were improvements in knee extensors and flexors and in hip extensors muscles. Ankle plantar flexors strength increased in half of the participants.	Most of the participants showed reduction smaller than 1s in Timed up and Go test (only one reduced 1.5s).		There were no differences in the emotional and behavioral tests.	
[28]		Participants showed significant improvements in PBS scores following dance protocol. There were not significant differences in quality of upper extremity skills test.			
[29]			Children reported high enjoyment level and desire for more classes. Parents reported perceived therapeutic benefits and therapist viewed the classes as a positive adjunct to therapy		
[32]		Significative improvements in GMFM scores dimensions D and E.	Although caregiver reported physical, cognitive, and emotional improvements following dance protocol, and COPM did not show significant changes.		
[5]	No significant changes we found for body composition, bony density, and hand muscle strength.	No significant changes we found for habitual physical activity and selective motor control of lower extremity, Time of ambulation decreased following dance protocol. There were significant differences in step length on right and stride length on left. There was improvement in inhibitory control with large individual response primarily among those above the mean at baseline.			
[17]			Significant improvements in domains of independence function, mainly mobility and communication in FIM. Improvements in body function, activity, and participation in WHODAS.		

**Table 4:** Quality appraisal for each included study.

Study	1	2	3	4	5	6	7	8	9	10	11	12	Classification
[33]	NA	NA	NA	NA	−	−	+	−	−	?	−	?	High risk of bias
[34]	+	+	+	−	+	+	NA	NA	+	−	+	?	High risk of bias
[26]	−	−	+	_−_	+	−	NA	NA	+	−	+	?	High risk of bias
[30]	NA	NA	NA	NA	−	+	+	−	+	−	+	?	High risk of bias
[27]	NA	NA	NA	NA	−	−	+	−	+	−	−	?	High risk of bias
[31]	NA	NA	NA	NA	−	−	+	−	+	−	+	?	High risk of bias
[9]	−	−	+	−	−	−	NA	NA	+	−	?	?	High risk of bias
[28]	−	−	+	?	−	−	NA	NA	+	?	+	?	High risk of bias
[29]	NA	NA	NA	NA	−	−	+	−	+	−	?	?	High risk of bias
[32]	NA	NA	NA	NA	−	−	+	−	+	−	−	?	High risk of bias
[5]	NA	NA	NA	NA	−	+	+	+	+	?	+	?	High risk of bias
[17]	+	+	+	−	+	+	NA	NA	+	?	−	?	High risk of bias

(1) random sequence generation, (2) allocation concealment, (3) baseline characteristics similar, (4) baseline outcomes similar, (5) confounding unlikely, (6) appropriate analyses (e.g., adjusted or time trend analyses if required), (7) sample representative of source population, (8) intervention independent of other changes, (9) intervention integrity (e.g., intervention delivered as intended and consistently, adequate protection against contamination), (10) blinding of outcome assessment, (11) incomplete outcome data addressed, and (12) free of other bias. NA: not applicable. (+): low risk of bias; (−): high risk of bias; (?): unclear.

**Table 5:** Evidence synthesis GRADE.

	Question: Dance Should Be Used in the Rehabilitation of Children and Adolescents with Neuromotor Disability?						
**Body Structure and Function Outcomes**	**Sample Size**	**Risk of Bias**	**Inconsistency**	**Indirecteness**	**Imprecision**	**Publication Bias**	**Overall Certainty of Evidence**
Postural sway [26,30,31]	23	Very serious ^a,b,c,d^	Not Serious	Serious ^e^	Very serious ^g^	Very serious ^h^	⨁◯◯◯ VERY LOW
Range of movement lower limbs [26,27]	23	Very serious ^a,b,c,d^	Not serious	Serious ^e^	Very serious ^g^	None	⨁◯◯◯ VERY LOW
Pain [9,26]	27	Very serious ^a,b,c,d^	Not serious	Very serious ^e^	Very serious ^g^	None	⨁◯◯◯ VERY LOW
Muscle strength lower limbs [26,31]	13	Very serious ^a,b,c,d^	Not serious	Serious ^e^	Very serious ^g^	None	⨁◯◯◯ VERY LOW
**Activity Outcomes**	**Sample Size**	**Risk of Bias**	**Inconsistency**	**Indirectenes**	**Imprecision**	**Publication Bias**	**Overall Certainty of Evidence**
Rhythm [26,30]	19	Very serious ^a,b,c,d^	Not serious	Not serious	Very serious v	Very serious ^h^	⨁◯◯◯VERY LOW
Gait [5,26,26]	31	Very serious ^a,c,d^	Not Serious	Serious ^e,f^	Very serious ^g^	None	⨁◯◯◯ VERY LOW
Attention [26,30]	19	Very serious ^a,b,c,d^	Not serious	Not serious	Very serious ^g^	Very serious ^h^	⨁◯◯◯ VERY LOW
Gross motor function [17,26,32,34]	53	Very serious ^a,b,c,d^	Not serious	Very serious ^e^	Very serious ^g^	None	⨁◯◯◯ VERY LOW
Funcional balance [28,30,34]	22	Very serious ^a,b,c,d^	Not serious	Serious ^e^	Very serious ^g^	None	⨁◯◯◯ VERY LOW
**Contextual Factors** **(Personal)**	**Sample Size**	**Risk of Bias**	**Inconsistency**	**Indirecteness**	**Imprecision**	**Publication Bias**	**Overall Certainty of Evidence**
[9,33]	28	Very serious ^a,b,c,d^	Not serious	Serious ^f^	Very serious ^g^	None	

Explanations: ^a^ Most studies did not apply or describe concealed allocation, blinded the therapists, applied intention to treat analysis, and reported missing data; ^b^ Differences in population (applicability); ^c^ Reviews that are based on small randomized controlled trials; ^d^ No description of missing data; ^e^ High heterogeneity between studies, even within subgroup analysis (high values for I^2^ or prediction intervals); ^f^ Differences in treatment intensity and content; ^g^ Small sample size; ^h^ Studies published by same research group.

## Data Availability

As a systematic Review, all the article data are showed in the paper.

## Supplementary Materials

The following supporting information can be downloaded at: https://www.mdpi.com/article/10.3390/ijerph20021501/s1.
