# Supplementary material for: Effectiveness and Evidence Level of Dance on Functioning of Children and Adolescents with Neuromotor Impairments: A Systematic Review
Source: Int J Environ Res Public Health. 2023 Jan 13;20(2):1501. doi: 10.3390/ijerph20021501 (PMC9859262; doi:10.3390/ijerph20021501)
Supplement: Supplementary file 1 [file ijerph-20-01501-s001.zip › ijerph-2026807-supplementary.pdf]

EFFECTIVENESS AND EVIDENCE LEVEL OF DANCE ON FUNCTIONING OF CHILDREN WITH NEUROMOTOR IMPAIRMENTS: A SYSTEMATIC REVIEW

Children and adolescents with NEUROMOTOR IMPAIMENTS

- ✓ Cerebral palsy;
- ✓ Developmental coordination disorders;
- ✓ Myelomeningocele;
- ✓ Syndromes with neuromotor impairments;
- ✓ Neurodegenerative diseases

Dance – Therapeutic Resource

12 STUDIES

ALL OF THEM WITH HIGH RISK OF BIAS

EVIDENCE

Very low for Body Structure and Function and Activity

- Small samples
- No randomized control groups
- No intention of treatment analysis
- Heterogeneity in intervention designs and measured outcomes

Most of the outcomes addressed ICF components:

- *body structure and function (n=7)*
- *Activity (n=11)*
- *personal factors (n=4)*
- *Participation (n=3)*

All the studies reported positive effects found in dance as a therapeutic tool

Rhythm · *Range of motion* · *Streight*

Postural sway · *Gait* · *Posture*

Pain · *Atention* · *Balance*

The LOW evidence level for dance on activity and body structure and function ICF domains does not mean that dance could not be use as adjuvant rehabilitation tool with potential benefits in participation and personal factors.

Further RCTs should assess the effects of dance in CANI using outcome measures in all components of the CIF
